# Beyond the Headaches: Severe Dysphagia As the Dominant Symptom of Spontaneous Intracranial Hypotension

**DOI:** 10.7759/cureus.94798

**Published:** 2025-10-17

**Authors:** Maheen Iqbal, Abdullah Shaik, Ayman Tahir, Saad Ansar, Reham Umer

**Affiliations:** 1 Acute Medicine, Peterborough City Hospital, Peterborough, GBR; 2 General Medicine, Peterborough City Hospital, Peterborough, GBR

**Keywords:** brain sagging, cerbellar herniation, magnetic resonance imaging, neurology, spontaneous intracranial hypotension (sih)

## Abstract

Spontaneous intracranial hypotension (SIH) is a rare but recognized condition that usually presents with orthostatic headache, brain sagging, and meningeal enhancement on an MRI. However, atypical presentations with a wide range of neurological symptoms are possible. Dysphagia is rarely documented as a dominant symptom in SIH. We present the case of a 50-year-old male patient with progressive slurred speech, facial weakness, gait instability, and severe swallowing dysfunction. Initial CT and MRI imaging revealed cerebellar tonsillar herniation and a left paravermian cyst, suggesting possible SIH in the background of a Chiari malformation. The definitive diagnosis of intracranial hypotension was made with brainstem sagging and meningeal enhancement seen on MRI with IV contrast. No evidence of a cerebrospinal fluid (CSF) leak was found on serial imaging, including a CT myelogram. The patient experienced severe and clinically significant pharyngeal dysphagia, confirmed on videofluoroscopy, with recurrent aspiration and the need for prolonged nasogastric feeding. Initially, he was managed with hydration, caffeine, and adequate bed rest without improvement. Input from neurology, neurosurgery, and SIH multidisciplinary (MDT) teams from tertiary centers was sought, with a plan for a blind lumbar epidural blood patch. However, the patient showed spontaneous clinical improvement over the course of admission and was discharged with outpatient neurology follow-up. This case illustrates the diagnostic challenges of SIH in the absence of demonstrable CSF leak and reinforces the consideration of SIH in the differential diagnosis of acquired Chiari-malformation-like radiological findings.

## Introduction

Intracranial hypotension has been a recognized post-traumatic or post-operative complication in the domain of neurosurgery since the early twentieth century [1]. Spontaneous intracranial hypotension (SIH) typically results from reduced cerebrospinal fluid (CSF) volume and pressure secondary to occult spinal CSF leakage. The incidence of SIH is very rare (three to five cases per 100,000 annually) and is usually experienced by adults with a female predominance [2]. The classic symptom is an orthostatic headache (i.e., worsening headache on standing but relieved by recumbence) [3]. SIH can also present atypically with cerebellar signs, cranial nerve deficits, cognitive changes, and dysphagia, thereby obscuring the diagnosis [4].

Widely varying manifestations of SIH often turn it into a diagnostic conundrum. In line with this, a UK survey comprising up to 64 patients with confirmed SIH reported that the patients needed to visit the general physician (GP) a median of three times before neurologist referral. Furthermore, up to 45% cases were not diagnosed at the first specialist consultation [5]. This can significantly impact the initiation of disease-specific treatment, which can be deferred for periods longer than 12 weeks [6]. This necessitates early identification of typical or atypical clinical signs of SIH. In addition, diagnosis also relies on neuroimaging features such as pachymeningeal enhancement, sagging of the brain, and tonsillar descent [7]. Nonetheless, acquired cerebellar tonsillar ectopia can mimic Chiari I malformation and may coexist with incidental posterior fossa arachnoid cysts, complicating interpretation. Moreover, accurate identification of a potential CSF leakage on spinal imaging can be arduous but remains critical to the overall SIH treatment [3].

We hereby describe a case which highlights SIH presenting predominantly with severe dysphagia along with acquired tonsillar ectopia and an incidental arachnoid cyst.

## Case presentation

A 50-year-old male patient presented with progressive slurred speech and left-sided weakness over three days. His family reported recent behavioral changes, confusion, unsteadiness, and weight loss. He had a three-week productive cough without fever. No other symptoms, including headaches, were reported. His past medical history was unremarkable, with no regular medications or allergies. He had no neurosurgical history and no previous spinal anesthesia or lumbar puncture (LP). Social history included cigarette and occasional cannabis use, and minimal alcohol.

On examination, the patient was alert but partially oriented. Glasgow coma scale (GCS) was 15/15, and speech was markedly slurred. Limb power was preserved, but gait was ataxic and unsteady, requiring assistance. Cranial nerve assessment revealed mild right facial asymmetry, lingual deviation to the right, and reduced palatal elevation. He didn’t have any nystagmus, dysmetria, any sensory loss or urinary incontinence. Swallowing assessment showed severe pharyngeal dysphagia with delayed initiation, reduced laryngeal elevation, and immediate coughing on all trials. He was kept nil by mouth with short-term nasogastric feeding recommended. Other systems were unremarkable except for moderate bilateral chest rhonchi. His vital signs remained stable.

**Figure 1:**
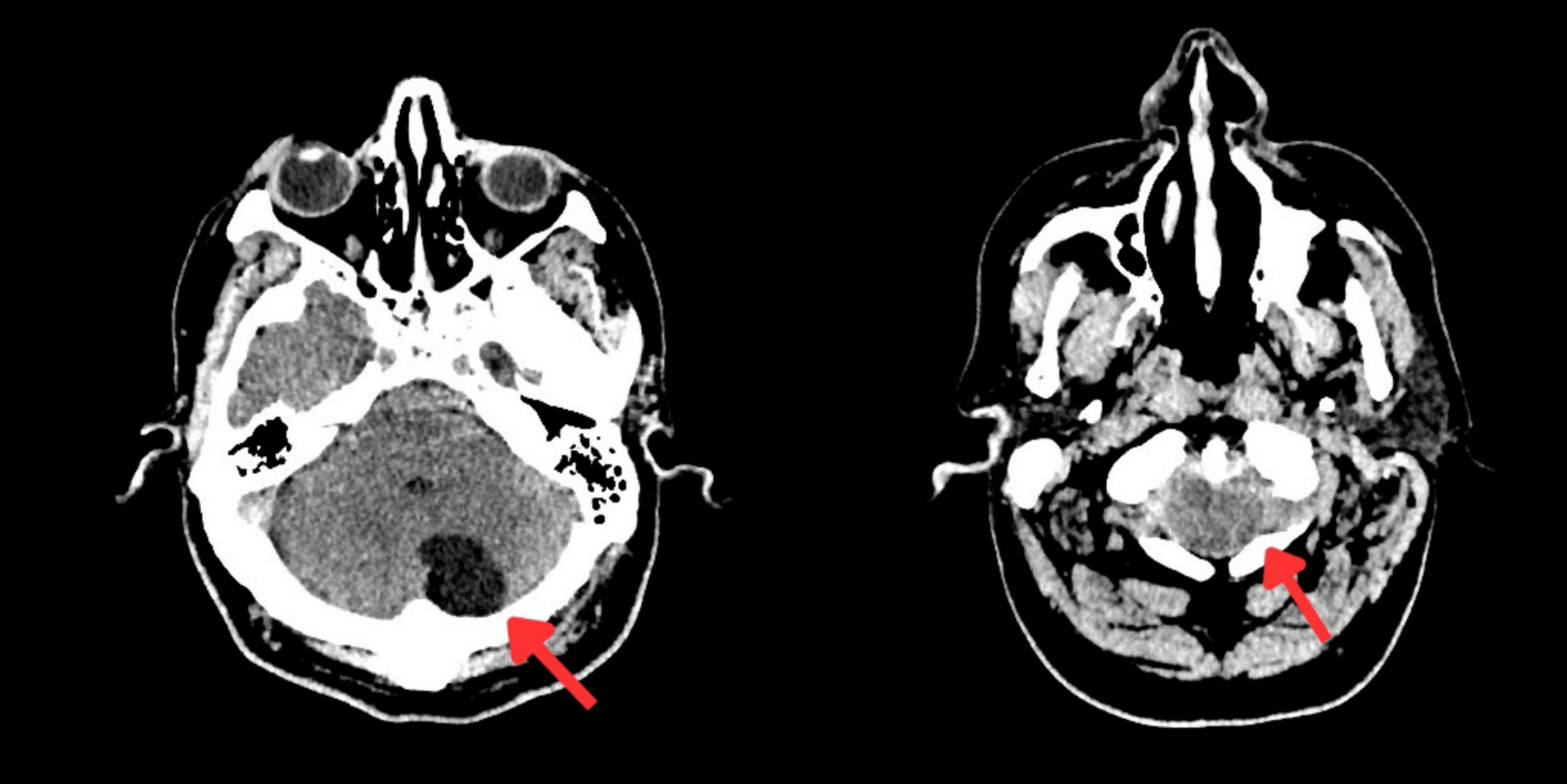
Non-contrast computed tomography (NCCT) head shows cerebellar cyst and tonsillar ectopia (indicated by arrows)

CT head (Figure 1) showed no acute infarct or hemorrhage, but revealed a left paravermian cerebellar cyst with cerebellar tonsillar ectopia.

**Figure 2:**
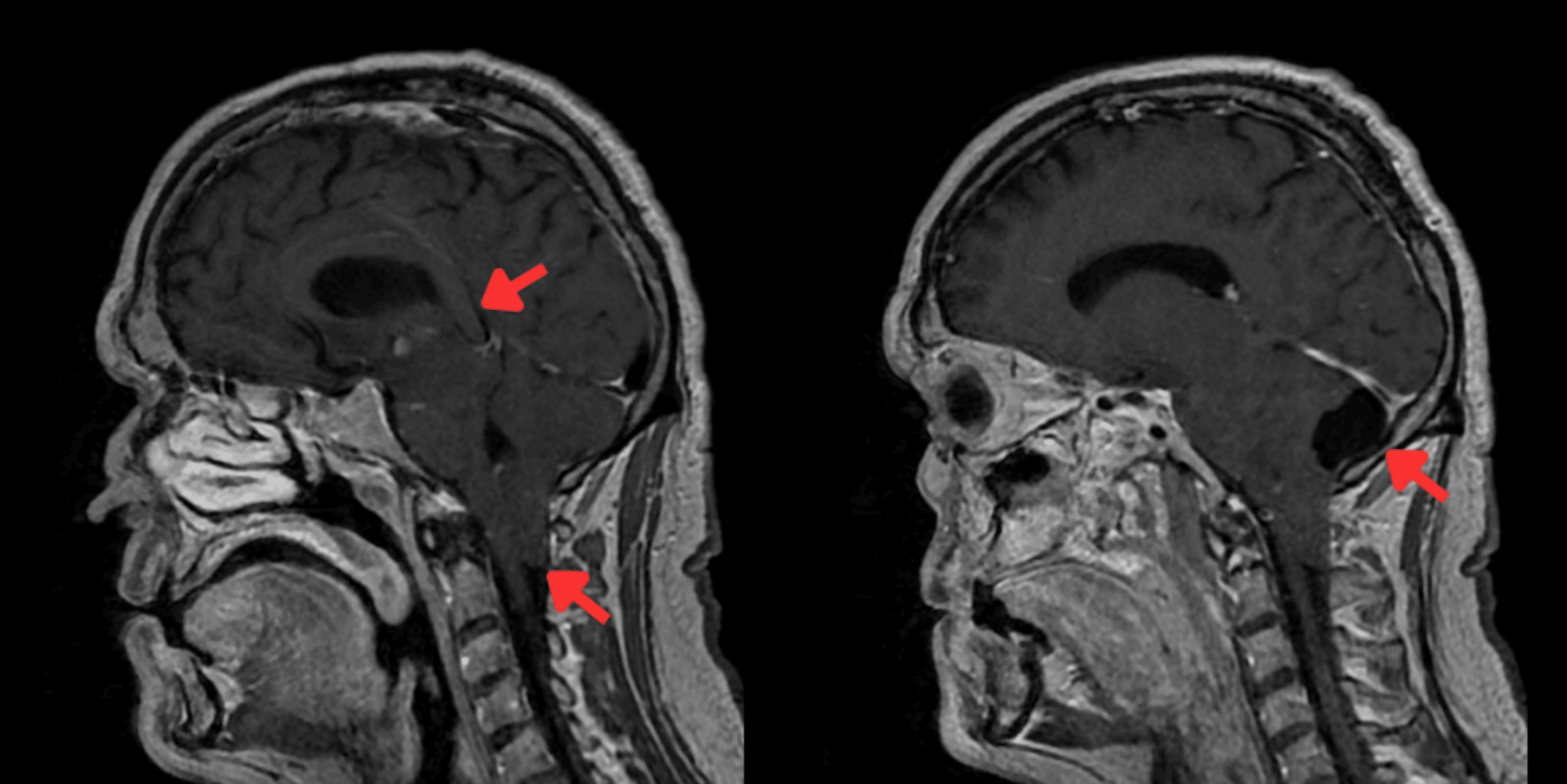
MRI head T1/T2 showing arachnoid cyst and tonsillar descent, brain sagging, and drooping of the corpus callosum (as indicated by arrows)

MRI head (Figure 2) with contrast demonstrated brainstem sagging, low-lying tonsils, and pachymeningeal enhancement consistent with SIH, along with a stable posterior fossa arachnoid cyst.

**Figure 3:**
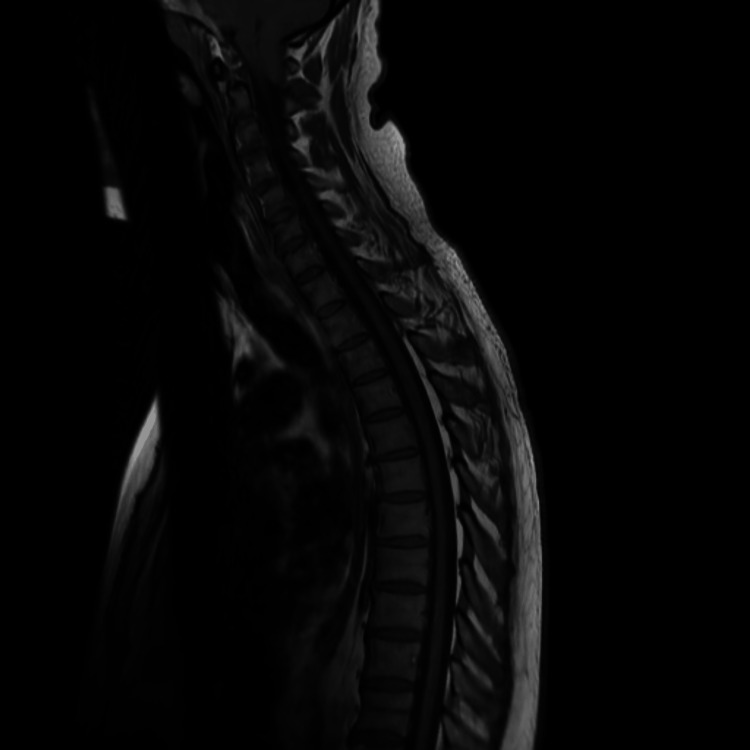
MRI whole spine showing no abnormalities

MRI spine (Figure 3) was unremarkable, showing no cord compression, bony abnormality, or CSF leak.

**Figure 4:**
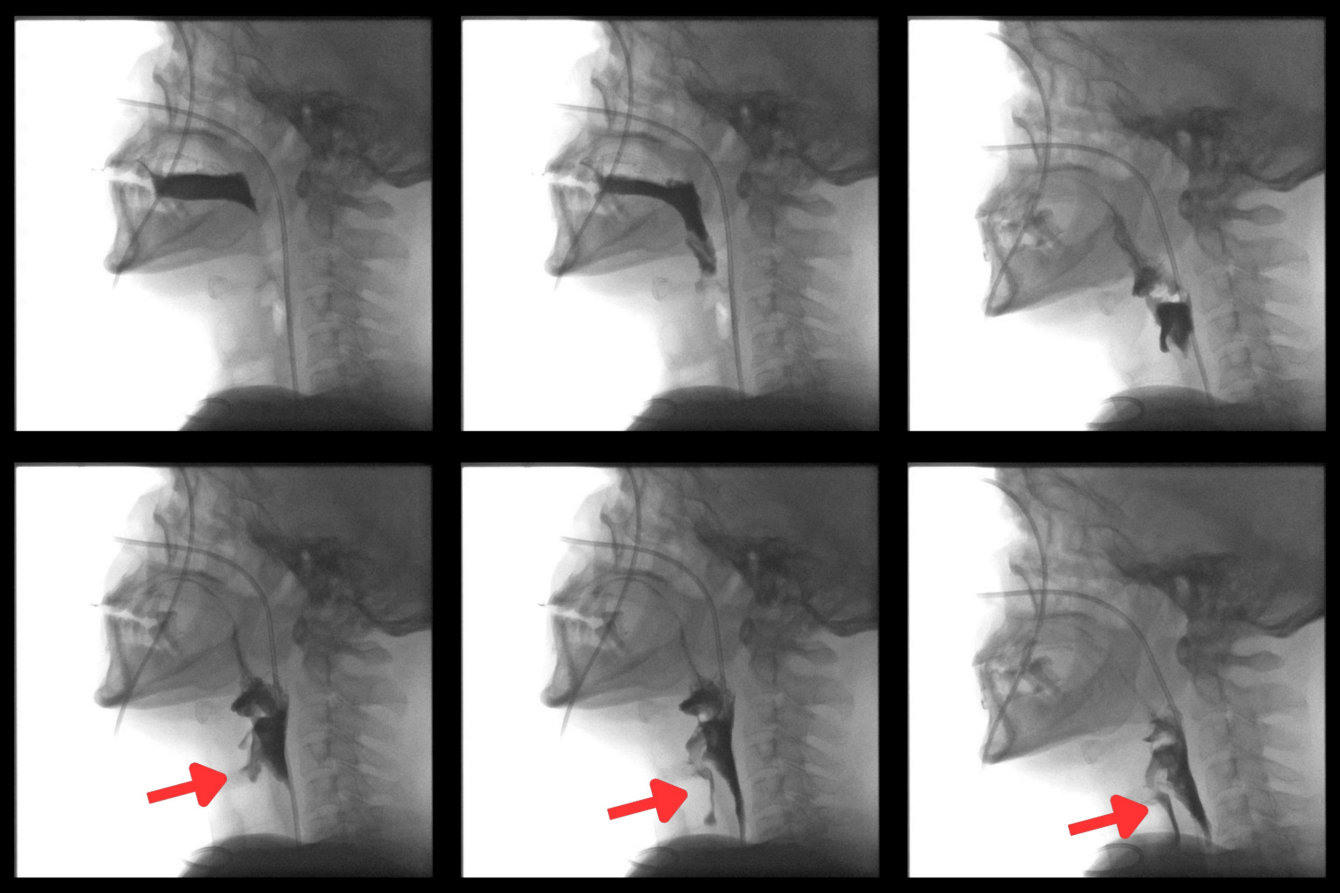
Video swallow study with arrows showing aspiration into the trachea No tools were used in this study as it was a radiological investigation.

Video swallow study (Figure 4) confirmed moderate to severe pharyngeal dysphagia with intermittent aspiration, particularly on thin and thick fluids, and the patient was deemed high-risk for aspiration.

Initially, conservative management with hydration, bed rest, and caffeine was instituted without improvement over two weeks. The speech and language therapy team recommended that the patient remain nil by mouth, allow only limited water sips, and maintain nutrition via nasogastric tube feeding. During hospitalization, the patient experienced partial improvement in dysphagia and functional status, with continued cognitive and gait disturbances. Neurology and neurosurgery teams were involved, and the SIH multidisciplinary team (MDT) review suggested consideration of a blind lumbar epidural blood patch. The patient was subsequently discharged since the symptoms improved drastically with conservative management and he was referred to Neurology as an outpatient. However, since the MRI whole spine (Figure 3) didn’t show any leakage, the epidural blood patch was not performed.

## Discussion

SIH can often remain underdiagnosed due to its atypical presentation secondary to bulbar symptoms stemming from brainstem traction [3,8]. Although an orthostatic headache is the chief clinical presentation documented in the most recent case studies from the literature (Table 1), our case demonstrates a mixed range of clinical symptoms ranging from focal neurological weakness to bulbar features, including dysphagia.

**Table 1:** Literature review of recent case reports detailing the management of SIH M: Male; MRI: Magnetic resonance imaging; CT: Computed tomography; SIH: Spontaneous intracranial hypotension; CSF: Cerebrospinal fluid; SDH: Subdural hematoma.

Authors	Presenting symptoms	Imaging (brain MRI/CT)	Treatment	Prognosis
Jurcau et al. (2024) [8]	51/M, admitted with 7-day orthostatic headache and horizontal diplopia (abducens palsies)	Brain MRI showed diffuse pachymeningeal enhancement and subtle brain sagging (stable posterior fossa cyst)	Conservative	Complete resolution; symptom-free at 1-year
Turnbull et al. (2021) [9]	59/F, presented with altered mental status after a fall, followed by a severe orthostatic headache	MRI head showed diffuse pachymeningeal enhancement, pronounced cerebellar tonsillar herniation	Administration of hypertonic saline, mannitol, and emergent posterior fossa decompression plus epidural blood patches	Gradual full recovery; normal MRI and neurologic exam at 5 months follow-up
Signorelli et al. (2024) [16]	32/M, presented with 2-month thunderclap headache (orthostatic) and horizontal diplopia	CT head showed bilateral chronic subdural hematomas, slit ventricles, and caudal descent of brainstem (tonsillar ectopia)	High-dose steroids; indomethacin also used as headache persisted	Complete resolution of headache and diplopia
Wu et al. (2023) [17]	66/M, admitted with severe right temporal orthostatic headache	Brain MRI showed no pachymeningeal enhancement or sag; spine MRI/myelography showed focal CSF collection at left T4–5 (later T5–6)	CT-guided targeted epidural blood patch at T4–5	Partial relief only; headache recurred; patient deteriorated and died secondary to aspiration pneumonitis
Goh et al. (2025) [10]	42/M, seen with an orthostatic headache and neck pain	Brain MRI showed bilateral chronic subdural hematomas; diffuse pachymeningeal enhancement (brain sag signs); spine MRI: suspected cervical CSF leak	Surgical drainage of SDH plus targeted cervical epidural blood patch treatment	Uneventful recovery

Typical SIH-related neuroimaging findings include diffuse pachymeningeal enhancement, downward descent of the midbrain and cerebellum, dural venous engorgement, subdural hygromas or hematomas, and less frequently, pituitary engorgement [9,10]. Comparably, our patient’s MRI showed diffuse leptomeningeal enhancement and cerebellar tonsil descent on sagittal views. These findings reflect compensatory meningeal vasodilation and downward traction when CSF buoyancy is lost [3,11]. Importantly, low-lying tonsils in SIH may mimic a Chiari I malformation. Nonetheless, a low-lying tonsil with pachymeningeal enhancement and orthostatic features should raise a strong suspicion of SIH [12]. Incidental posterior fossa lesions (e.g., arachnoid cysts) can further confound MRI interpretation. In our case, the posterior fossa arachnoid cyst was likely incidental, but contributed to initial concern for a structural lesion. In contrast to brain imaging, spinal imaging to localize a CSF leak can be significantly challenging. An MRI of the spine is often unrevealing, and more dynamic imaging studies, e.g., CT or MR myelography or digital subtraction myelography, may be needed to isolate a slow-flow CSF leakage. In our patient, the spinal MRI was unremarkable, and no leak was found on the first myelography, thereby reflecting how elusive leaks can be.

Initial management of SIH is supportive, mainly focusing on strict recumbence, hydration, and analgesics [10,12]. If headaches persist, the mainstay is an epidural blood patch, where autologous blood is injected epidurally to seal the leak. Moreover, blind lumbar epidural blood patches often provide relief in many patients [12,13]. For refractory cases or once MRI imaging has localized the leak, targeted epidural blood patches at the leak level or even surgical repair may be performed.

Fortunately, the prognosis of SIH is generally satisfactory with treatment, where most patients recover following one to two epidural blood patches [12]. The overall recovery process, however, can be more short-term (88%) as compared to long-term improvement (33%) [14]. The latter also reported that 36 out of 51 (71%) patients had a continuous spinal CSF leakage on the post-epidural patch imaging [14]. Patients who remain undiagnosed or untreated are at a higher risk of persistent disability, even coma from herniation or seizure, and in rare cases, can have poor outcomes [15]. Early recognition of the orthostatic headache and other clues is thus, crucial. Clinicians should maintain a high suspicion for SIH in patients with unexplained bulbar symptoms, even without orthostatic headache or detectable CSF leak. Imaging correlation and careful follow-up are critical for diagnosis and avoiding unnecessary surgical interventions.

## Conclusions

Severe dysphagia can present as the dominant and functionally debilitating manifestation of SIH, even in the absence of the typical orthostatic headache or a visible CSF leak. In such cases, acquired cerebellar tonsillar descent may mimic a Chiari I malformation, and incidental posterior fossa findings such as arachnoid cysts should be interpreted within the clinical context.

Early recognition of these atypical presentations and multidisciplinary collaboration between neurology, neuroradiology, rehabilitation, and speech and language therapy teams are crucial for timely diagnosis and optimal management. Even when CSF leakage is not visualized, appropriate supportive care and follow-up can lead to significant symptomatic improvement and prevent long-term morbidity.
